# Dynamic Neuroimaging Biomarkers of Smoking in Young Smokers

**DOI:** 10.3389/fpsyt.2020.00663

**Published:** 2020-07-10

**Authors:** Ting Xue, Fang Dong, Ruoyan Huang, Zhanlong Tao, Jun Tang, Yongxin Cheng, Mi Zhou, Yiting Hu, Xiaojian Li, Dahua Yu, Haitao Ju, Kai Yuan

**Affiliations:** ^1^ School of Science, Inner Mongolia University of Science and Technology, Baotou, China; ^2^ Inner Mongolia Key Laboratory of Pattern Recognition and Intelligent Image Processing, School of Information Engineering, Inner Mongolia University of Science and Technology, Baotou, China; ^3^ Department of Neurosurgery, Affiliated Hospital of Inner Mongolia Medical University, Hohhot, China; ^4^ Life Sciences Research Center, School of Life Science and Technology, Xidian University, Xi’an, China

**Keywords:** dynamic, amplitude of low-frequency fluctuation (ALFF), young smokers, resting-state functional magnetic resonance imaging (rs-fMRI), regional homogeneity (ReHo)

## Abstract

**Objective:**

To examine potential changes in the dynamic characteristics of regional neural activity in young smokers and to detect whether the changes were associated with smoking behavior.

**Methods:**

The dynamic regional homogeneity (dReHo) and dynamic amplitude of low-frequency fluctuations (dALFF) in 40 young smokers and 42 nonsmokers were compared. Correlation analyses were also performed between dReHo and dALFF in areas showing group differences and smoking behavior [e.g., the Fagerström Test for Nicotine dependence (FTND) scores and pack-years].

**Results:**

Significantly differences in dReHo variability were observed in the inferior frontal gyrus (IFG), superior frontal gyrus (SFG), medial frontal gyrus (MFG), insula, cuneus, postcentral gyrus, inferior semi-lunar lobule, orbitofrontal gyrus, and inferior temporal gyrus (ITG). Young smokers also showed significantly increased dALFF variability in the anterior cingulate cortex (ACC) and ITG. Furthermore, a significant positive correlation was found between dALFF variability in the ACC and the pack-years; whereas a significant negative correlation between dReHo variability in the IFG and the FTND scores was found in young smokers.

**Conclusion:**

The pattern of resting state regional neural activity variability was different between young smokers and nonsmokers. Dynamic regional indexes might be a novel neuroimaging biomarker of smoking behavior in young smokers.

## Introduction

There are more than one billion smokers globally according to the report in 2017 published by the Word Health Organization (https://www.who.int/). It has been demonstrated that the age period from late adolescence to adulthood is a crucial period of time of continued brain development (1). Numerous findings showed that people at this age have high prevalence of smoking cigarettes, and people who start smoking cigarettes at this age is more susceptible to nicotine addiction (2, 3). Therefore, the elucidation of neural mechanisms underlying smoking cigarettes in young smokers might be helpful for preventing nicotine addiction.

Although the exact mechanism of nicotine addiction is still unknown, recent neuroimaging findings have advanced our understanding (4–8). Resting-state functional magnetic resonance imaging (rs-fMRI), measuring task-independent spontaneous brain activity in low-frequency blood oxygenation level dependent (BOLD) fluctuations (9), has been widely applied for delineating functional neural abnormalities in smokers (10–17). Previous studies have demonstrated that regional spontaneous neural characteristics are very important for understanding neuropathological and neurophysiological conditions (18). For example, abnormal regional energy consumption indicates decreased or excessive resting metabolic rates (19). Currently, the widely used approaches for characterizing spontaneous regional neural properties are the regional homogeneity (ReHo) and amplitude of low-frequency fluctuation (ALFF). Specifically, ReHo measures the temporal synchronization of regional neural activity among spatially adjacent regions (20); whereas ALFF reflects the intensity of regional neural activity (21). These two approaches have been widely applied to evaluate regional neural function in neuropsychiatric diseases and neurologic disorders (21–24).

In the context of nicotine addiction, numerous rs-fMRI studies have focused on the properties of regional neural activity (12, 25–28). For example, compared with nonsmokers, Yu and his colleague discovered that heavy smokers exhibited decreased ReHo in the prefrontal regions and increased ReHo in the insula and posterior cingulate cortex (PCC) (12). Another study found decreased ReHo in the inferior frontal cortex and increased ReHo in the superior parietal lobe (25). Chen et al. further found decreased ReHo in the superior frontal gyrus (SFG) and precuneus, and the decreased ReHo value in the precuneus was positively correlated with the Fagerström Test for Nicotine dependence (FTND) scores, an important index for clinical evaluation of smoker’s dependence on nicotine addiction (26). Using fractional ALFF technology, enhanced neural spontaneous activity was found in the caudate of young smokers (28). Another study focused on regional spontaneous neural activity intensity in heavy smoker and discovered decreased spontaneous activity in the precuneus and inferior temporal gyrus (ITG) and increased spontaneous activity in the caudate (27). These findings indicated that static ReHo and ALFF were different at rest between smokers and nonsmokers.

However, the above-mentioned investigations ignored the dynamic characteristics of spontaneous neural activity, and they usually assumed the BOLD signal during scanning was stationary. In fact, it has been suggested that the brain dynamically responds and adjusts to external or internal stimuli over multiple time-scales (29, 30). By characterizing temporal changes in the brain’s resting state functional connectivity, the intrinsic neural activity was also found to be time-varying in nature (31–34). Moreover, high-resolution spatiotemporal functional neuroimaging studies have confirmed substantial temporal variations in regional neural activity (35, 36). Consequently, elucidation of the temporal variability in oscillation amplitudes and synchronization of regional neural activity may help us better understand the effects of smoking cigarettes on brain function. Fortunately, the time-varying characteristics of resting state regional neural activity may be examined by the sliding window approach. This approach has been shown to work well for evaluating dynamic properties of regional neural activity (37), and it has been widely applied due to its simplicity and ease of implementation (30, 38).

Taken together, considering there are substantial temporal fluctuations in regional neural activity (35), and until now, there have been very few studies concerned with the dynamic characteristics of regional neural activity in young smokers. Currently, a sliding window technology combined with the dynamic ALFF (dALFF) and dynamic ReHo (dReHo) approaches were applied to investigate whether young smokers would present with abnormal dynamic characteristics of regional neural activity. In addition, correlation analyses were also performed to ascertain the relationships between the altered regional indexes (the dALFF and dReHo) and smoking behavior. We hypothesized that the temporal characteristics of regional neural activity may be altered in young smokers, and dynamic indexes may be associated with smoking behavior. It is hoped that the present study advances our understanding of nicotine addiction.

## Materials and Methods

### Ethics Statement

The present study was approved by the Ethical Committee of Medical Research in First Affiliated Hospital of Baotou Medical College, Inner Mongolia University of Science and Technology. All study procedures were conducted in accordance with the Declaration of Helsinki. After explaining the aims and steps in the procedure of the current study, all the participants and their guardians signed written informed consents.

### Subjects

The potential participants were male undergraduates from local universities recruited through advertisements. To screen for potential participants, a semistructured interview and questionnaires were used to assess psychiatric conditions, medical conditions, medication use, history of claustrophobia, history of substance use, and the presence of metal implants in their body. The inclusion criteria for smokers were 1) meeting DSM-V criteria for nicotine dependence, 2) reporting smoking ≥ 10 cigarettes per day in the last 6 months, 3) carbon monoxide (CO) > 6 parts per million (p.p.m.) in expired air (Smokelyzer, Bedfont Scientific, Kent, UK), and 4) having no period of smoking abstinence longer than 6 months in the past few years. We used the FTND and pack-years to assess nicotine dependence (39). Specifically, the FTND measured the severity of nicotine dependence (40), and a pack-years was defined as twenty cigarettes smoked per day for one year. The nonsmokers were included as those who smoked less than five cigarettes in their lifetime. Additional requirements for nonsmokers were CO concentrations below 3 p.p.m. in expired air. Exclusion criteria for both groups were 1) any physical illness such as epilepsy, brain tumor, obstructive lung disease, or hepatitis, 2) urine test revealing any other substance or drug abuse (except nicotine), 3) existence of a neurological disease, or 4) claustrophobia. A total of 520 potential subjects completed the semi-structured interview and questionnaires, and among them, 46 participants were recruited in the young smoker group. The incidence was 8.8%. Forty-eight nonsmokers were recruited in healthy controls group. All participants were right-handed and 17–24 years of age. This study only focused on differences in regional neural activity between nondeprived young smokers and nonsmokers. Therefore, young smokers were asked to refrain from smoking in the 30 min immediately preceding the scan to exclude withdrawal symptoms (41).

### Data Acquisition

The study was performed on a 3T magnetic resonance imaging (MRI) scanner (Achieva, Philips Healthcare, Best, the Netherlands) equipped with an 8-channel SENSE coil. The scanning parameters were repetition time (TR) = 2,000 ms, echo time (TE) = 30 ms, slice thickness = 5 mm, number of slices = 30, flip angle = 90°, field of view (FOV) = 220 mm × 220 mm, voxel size = 3mm × 3 mm × 3mm, matrix = 64 × 64, and 180 volumes. During the entire scanning procedure, a standard head coil and foam pads were used to minimize head movement. All subjects were instructed to close their eyes and stay still during scanning.

### Data Analysis

The rs-fMRI data were preprocessed using the Data Processing Assistant for Resting-State fMRI Analysis Toolkit (DPARSF). The details included 1) deleting the first 10 images, 2) correcting slice-time delay, 3) realigning to correct for head motion, 4) normalizing to the standard Montreal Neurological Institute (MNI) template provided by SPM using linear and nonlinear transformations, 5) resampling (3 mm × 3 mm × 3mm), and 6) regressing out nuisance covariates (24 head motion parameters, white matter signal, cerebrospinal fluid signal and linear trend). The head motion of all participants was examined. If the head motion displacement exceeded 2.0 mm or rotation exceed 2.0°, the corresponding data will be excluded from the current study. Six nonsmokers and six young smokers were excluded because of excessive head motion. Eventually, 40 young smokers and 42 nonsmokers were included in further analysis. Group differences in the mean framewise displacement (FD) were also compared. The results showed that there were no significant group differences (*p* = 0.781) between the two group groups. Additionally, each participant’s mean FD was included as a covariate in the follow-up, group-level analysis to reduce the effect of head movement. For ReHo calculations, the data were further filtered using a bandpass temporal filter of 0.01–0.08 Hz to reduce noise and low frequency drift.

The dALFF and dReHo analyses were performed using Temporal Dynamic Analysis (TDA) toolkits based on DPABI (42). Specifically, a temporal rectangular window was first chosen. Then, the ALFF and ReHo values in each window were calculated. Previous window-based analyses usually adopted a window length as small as 10 s (43) and as long as 180 s (44, 45). Ideally, the window size should be small enough to detect potentially transient signals, and yet large enough to analyze the lowest frequencies of interest in signals (45, 46). To avoid the introduction of spurious fluctuations, the minimum window length should be larger than 1/*f*_min_, where *f*_min_ is the minimum frequency of time series (47). Since there was currently no formal consensus regarding the window length, here a window length of 32 TR was selected according to previous studies (45), which was considered as the optimal parameter to maintain the balance between capturing a rapidly shifting dynamic temporal characteristics and obtaining reliable estimates of the regional neural activity (48). The preprocessed data of each individual were segmented into 139 windows. The ALFF and ReHo indexes were calculated in each window. In detail, the ReHo index was calculated using the Kendall’s coefficient of concordance (KCC) to characterize the similarity of the time series of a given voxel and its 27 neighboring voxels (20). The ALFF index was calculated as the amplitude interval over a frequency range of 0.01–0.08 Hz, reflecting the intensity of spontaneous neural activity (21). To obtain the dALFF and dReHo maps, the standard deviation (SD) of ReHo and ALFF values at each voxel across all windows were calculated. Then, the obtained dReHo and dALFF maps were further normalized by dividing dReHo and dALFF maps by the mean dReHo and dALFF values. Finally, an isotropic Gaussian kernel of 4 mm full-width-at-half-maximum (FWHM) was used to smooth the maps.

### Statistical Analysis

Group comparisons of demographic data were performed using two-sample *t* tests (*P* < 0.05). Group analyses of dALFF and dReHo maps were performed using the general linear model (GLM) in SPM12 with mean FD as a covariate. The results were thresholded at *P* < 0.001 (uncorrected) and a cluster size of 50 voxels, corresponding to a familywise error (FWE) correction for multiple comparisons of *P*_FWE_ < 0.05. Correlation analyses were conducted in young smokers between smoking behavior (FTND and pack-years) and mean dReHo/dALFF values in regions showing group differences with mean FD as a covariate. In the current study, the potential relationship between dynamic regional indexes and smoking behavior were surveyed in an exploratory investigation. Therefore, the threshold for statistical significance was set at *P* < 0.05 (uncorrected).

## Results

### Smoking Behavior

Forty young male smokers and 42 male nonsmokers were included in the current study. The detailed demographic information was presented in **Table 1**.

**Table 1:** Demographic and smoking behavior.

	Young smokers (*n* = 40)	Nonsmokers (*n* = 42)
Age, years	19.70 ± 2.02	19.05 ± 1.72
sex(M/F)	40/0	42/0
FTND	6.73 ± 1.48	–
pack-years	2.44 ± 0.93	–

Data represent mean ± standard deviation; M, male; F, female; FTND, Fagerström Test for Nicotine Dependence; Pack-years, Years of smoking × Cigarettes smoked per day/20.

### Group Differences in dReHo and dALFF Maps

**Figure 1:**
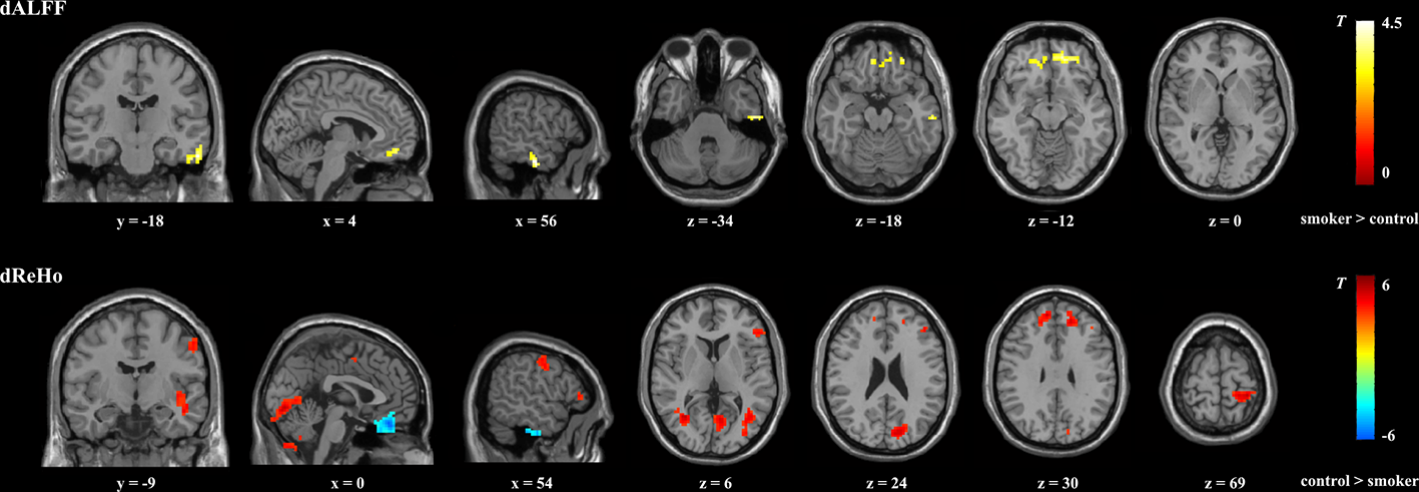
Brain regions showing significant group differences in dALFF and dReHo between young smokers and nonsmokers using familywise error-corrected analysis (*P_FWE_* < 0.05 with a minimum cluster size of *k* = 50 voxels and a cluster-defining threshold *P* < 0.001). dALFF, dynamic amplitude of low-frequencey fluctuation; dReHo, dynamic regional homogeneity.

Compared with nonsmokers, young smokers demonstrated significantly decreased dReHo variability in the inferior frontal gyrus (IFG), medial frontal gyrus (MFG), SFG, inferior semi-lunar lobule, postcentral gyrus, cuneus, and insula, whereas significantly increased dReHo variability was observed in the orbitofrontal gyrus and ITG (**Figure 1** and **Table 2**). Relative to the nonsmoker group, young smokers showed significantly increased dALFF variability in the ITG and anterior cingulate cortex (ACC) (**Figure 1** and **Table 3**).

**Table 2:** Brain regions showing significant differences in dynamic ReHo between groups.

			MNI coordinates				
	Brain region	BA	X	Y	Z	Cluster size	Peak *t* value
HC > Smoker	Inferior semi-lunar lobule	–	-9	-72	-51	660	6.25
	Cuneus	18/30	12	-81	24	502	5.71
	Postcentral gyrus	5	30	-36	69	130	5.16
	Inferior frontal gyrus	46	51	33	6	62	4.9
	Superior frontal gyrus	8/9	-12	51	30	90	4.87
	Medial frontal gyrus	8	15	42	33	74	4.77
	Insula	13	48	-9	-12	69	4.4
HC < Smoker	Orbitofrontal gyrus	11	0	39	-24	141	6.19
	Inferior temporal gyrus	20	51	-15	-39	60	5.72

ReHo, regional homogeneity; MNI, Montreal Neurological Institute; BA, Brodmann’s area; HC, healthy controls.

**Table 3:** Brain regions showing significant differences in dynamic ALFF between groups.

			MNI coordinates				
	Brain regions	BA	X	Y	Z	Cluster size	Peak *t* value
HC < Smoker	Inferior temporal gyrus	21	57	-18	-27	50	4.82
	Anterior cingulate cortex	32	15	45	-12	141	4.72

ALFF, amplitude of low frequency fluctuation; MNI, Montreal Neurological Institute; BA, Brodmann’s area; HC, healthy controls.

### Correlation Analysis

**Figure 2:**
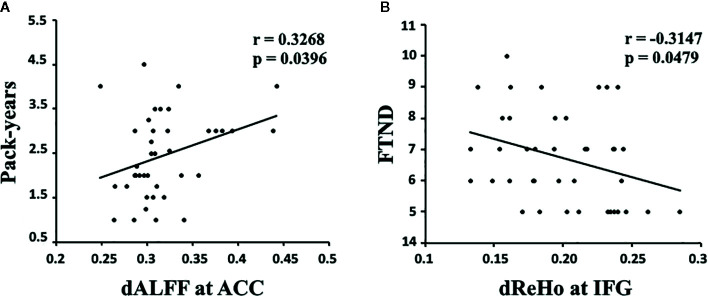
**(A)** The dALFF variability in the ACC was positively correlated with pack-years in young smokers (*r* = 0.3268, *p* = 0.0396, uncorrected). **(B)** The dReHo variability in the IFG was negatively correlated with FTND scores in young smokers (*r* = -0.3147, *p* = 0.0479, uncorrected). dALFF, dynamic amplitude of low-frequency fluctuation; dReHo, dynamic regional homogeneity; ACC, anterior cingulate cortex; IFG, inferior frontal gyrus; FTND, Fagerström Test for Nicotine dependence. Pack-years of smoking were calculated by multiplying the average number of packs of cigarettes smoked per day by the number of years the participant smoked.

The results showed that the temporal variability in dALFF in the ACC was positively correlated with pack-years (*r* = 0.3268, *p* = 0.0396, uncorrected; **Figure 2A**), and the temporal variability in dReHo in the IFG was negatively correlated with FTND (*r* = -0.3147, *p* = 0.0479, uncorrected; **Figure 2B**).

## Discussion

Previous studies have probed static regional neural activity in smokers. However, to the best of our knowledge, very few studies have tried to assess nicotine-related dynamic regional neural activity changes in young smokers. In the current study, dynamic regional neural activity between young smokers and nonsmokers was examined using sliding window analysis. Differences in dReHo variability were observed in the IFG, SFG, MFG, insula, cuneus, postcentral gyrus, inferior semi-lunar lobule, orbitofrontal gyrus, and ITG, whereas differences in dALFF variability in the ACC and ITG distinguished young smokers from nonsmokers. Further correlation analyses showed that the pack-years/FTND scores were related to abnormal dALFF variability in ACC/dReHo variability in IFG in young smokers. The current findings about the dynamic neural characteristics in young smokers may expand our understanding of nicotine-related neural mechanisms.

The aberrant reward function is believed to play a key role in the progress of addiction. To date, the findings in this field showed that subcortical regions are crucial to facilitating such progress (49, 50). However, recent studies indicated that the frontal-cingulate regions are also heavily involved in addiction progress (51). For example, the ACC was found to be hyperactivated to drug-related reward and hypoactivated to natural rewards (52, 53). Recent studies further confirmed the function of ACC linking reward to goal-directed behaviors (54). One study focused on substance dependence found that compared with drug-related stimuli, substance-dependent individual exhibit decreased reward to stimuli related to monetary gains. They speculated that monetary rewards may be devalued relative to drug-related reward, and such aberrant reward function may be related to abnormal goal-directed behavior (55). In the context of nicotine addiction, studies have shown that nicotine-related stimuli can elicit overvalued rewards than nonnicotine stimuli in abstinent smokers. This finding may reflect that the ACC would assign a larger reward value to nicotine-related behaviors in a state of craving (56, 57). The current findings of increased ALFF temporal variability in ACC further confirmed the abnormal function of the ACC in young smokers. Considering the importance of ACC in evaluating reward value related to goal-directed behaviors, we tend to speculate that the abnormal temporal characteristics of dALFF in the ACC might reflect reward processing impairment in young smokers, which may facilitate action-selection mechanisms to converge on nicotine addiction.

Substantial evidence demonstrated that the ACC and prefrontal regions are primary nodes of a putative “cognitive control network” (58). Specifically, the ACC plays an important role in detecting salient events, especially to erroneous or error-prone actions (59). Once detecting such events or actions, the ACC further signals reorientation of attention implemented by the prefrontal regions (60). Previous studies have indicated that abnormalities in the ACC and prefrontal regions were associated with impaired cognitive function (executive, inhibitory, and decision-making) in smokers (61, 62). Abnormal neural activity in these regions in smokers during abstinence may suggest inefficient cognitive control in abstinent smokers (63). Taken together, the abnormal dALFF and dReHo in the ACC and prefrontal regions and correlations between the changes in dynamic regional indexes and smoking behavior may provide novel neuroimaging biomarkers of smoking in young smokers.

The current study has several limitations. First, the sample size was small. Larger samples will be needed in future studies. Second, physiological noise from respiratory cycles and cardiac activity were not monitored during fMRI scanning. Finally, the current findings are cross-sectional, and longitudinal studies are needed to elucidate the dynamic characteristics of regional neural activity.

## Conclusion

To the best of our knowledge, very few studies have tried to assess dynamic regional neural activity in young smokers. We found increased dynamic ALFF variability in the ACC and decreased ReHo variability in frontal regions. Correlation analyses showed that the changes were related to the severity of nicotine addiction. This finding is indicative of the importance of cingulate and frontal regions in nicotine addiction in young smokers. Dynamic regional indexes might be used as a powerful supplement to static regional indexes, helping us obtain a more comprehensive understanding of neural activity in young smokers.

## Data Availability Statement

The datasets presented in this article are not readily available because of the privacy of all the participants. Requests to access the datasets should be directed to fmydh@imust.edu.cn.

## Ethics Statement

The studies involving human participants were reviewed and approved by Ethical Committee of medical research in First Affiliated Hospital of Baotou Medical College, Inner Mongolia University of Science and Technology. Written informed consent to participate in this study was provided by the participants’ legal guardian/next of kin.

## Author Contributions

TX, DY, and KY were responsible for the study concept and design. FD, RH, YH, MZ, ZT, JT, XL, HJ, and YC contributed to the acquisition of fMRI data. TX and RH performed the data analysis. DY and KY provided critical revision of the manuscript for important intellectual context.

## Funding

This work is supported by the National Natural Science Foundation of China under Grant Nos. 81871430, 81871426, 61771266, 81401488, 81701780, 31800926, 81401478, 81401488,81470816, and 81471737, the program for Young Talents of Science and Technology in Universities of Inner Mongolia Autonomous Region NJYT-17-B11, the Natural Science Foundation of Inner Mongolia under Grant No. 2019JQ07, the science and technology planning project of Inner Mongolia Autonomous Region 2019GG109, the Chunhui Program of the Ministry of Education of the People’s Republic of China 2018-45, the Fundamental Research Funds for the Central Universities under Grant No. JB151204, the Natural Science Basic Research Plan in Shaan-xi Province of China under Grant No. 2018JM7075, and the US National Institutes of Health, Intramural Research program Y1AA3009.

## Conflict of Interest

The authors declare that the research was conducted in the absence of any commercial or financial relationships that could be construed as a potential conflict of interest.
